# Motivational Incongruence and Well-Being at the Workplace: Person-Job Fit, Job Burnout, and Physical Symptoms

**DOI:** 10.3389/fpsyg.2016.01153

**Published:** 2016-08-11

**Authors:** Veronika Brandstätter, Veronika Job, Beate Schulze

**Affiliations:** ^1^Department of Psychology, University of ZurichZurich, Switzerland; ^2^Institute of Social Medicine, Occupational Health and Public Health, University of LeipzigLeipzig, Germany

**Keywords:** implicit affiliation motive, implicit power motive, job characteristics, burnout, occupational health

## Abstract

Person–environment fit has been identified as a key prerequisite for employee well-being. We investigated to what extent a misfit between motivational needs and supplies at the workplace affects two key health outcomes: burnout and physical symptoms. Individual needs (implicit affiliation and power motives) and environment supplies (motive specific job characteristics) were assessed in an online survey of full time employees (*n* = 97), using a picture story exercise measuring implicit motives and a scale listing affiliation and power related job characteristics. Outcomes were assessed using the Maslach Burnout Inventory and a checklist of physical symptoms. We conducted polynomial regressions with response surface analysis. Results reveal that motivational incongruence with respect to the affiliation motive was related to high job burnout, while motivational incongruence concerning the power motive predicted increased physical symptoms. This was true for both those with a strong affiliation or power motive and low corresponding job characteristics and those with a weak affiliation or power motive and job characteristics demanding the respective motive. Results hint at potential interventions toward preventing or remedying a lack of needs-supply fit and reducing the risk of impairments of well-being.

## Introduction

Imagine an accountant who actually is an outgoing person, enjoys being in company and seeks closeness in her social relationships. However, at her workplace, she most of the time works on her own with hardly any contact with colleagues or clients. Thus, her job does not offer many opportunities to socialize and to be in a trusting mutual exchange with other people. And now imagine another employee, a mid-level manager, who is expected to take on responsibility for his team, motivate and supervise his staff members, find compromises between conflicting interests, make personnel decisions, in short, to influence on other people. When at his workplace, though, he is out of his element as he does not like to take center stage and actually feels awkward in his role as a leader. As different, at first sight, the situation of these two employees might seem, there is one commonality between them: their motivational propensities with respect to the two social motives, namely affiliation and power, do not match with the demands and opportunities their job offers them, that is, a motivational person–environment misfit exists. Would employees’ emotional well-being and health be affected by the motivational incongruence described?

Indeed, following a conception advanced in the organizational literature on work stress and job burnout under the heading of *person–environment fit* (Edwards and Shipp, 2007; Krumm et al., 2013) or *job-person match* (Maslach et al., 2001; Maslach and Leiter, 2008) one would have to answer the question in an affirmative way. This theoretical notion of a person–environment interaction is in line with the basic tenet of motivation psychology that is expressed most succinctly in Kurt Lewin’s famous statement that human experience and behavior “is a function of the person and of his environment” (Lewin, 1951, p. 239). There is no doubt in motivation research that emotional experience and goal directed behavior result from an interplay of characteristics of the person and the environment. Surprisingly, only few theoretical accounts and, consequently, only a few studies have tested this question empirically (Hull, 1943; Atkinson, 1957; Andrews, 1967; Jenkins, 1987; Brunstein and Maier, 2005; Schattke et al., 2014; see also Stanton et al., 2010). The majority of studies on motivation either focuses on individuals’ (e.g., basic needs, motives, goals, interest, values; Scheffer and Heckhausen, 2008) or on situational characteristics (e.g., social context, incentives, opportunities for acting, subconscious priming cues; Beckmann and Heckhausen, 2008; Bargh et al., 2010) as determinants of affect, thought, and behavior.

The present research makes a case for an analysis of the interaction between within-person implicit motives and situation-specific behavioral opportunities (i.e., motive relevant incentives) in an individual’s environment. More concretely, we aim at assessing whether a misfit between an employee’s implicit affiliation and power motives (person) and the respective motivationally relevant behavioral opportunities associated with an employee’s occupational activities (environment) is detrimental to their emotional and physical well-being.

### Person-Environment Fit, Job Burnout, and Physical Symptoms

On the most general level, person–environment (P-E) fit has been conceptualized as “the congruence, match, similarity, or correspondence between the person and the environment” (Edwards and Shipp, 2007, p. 211) that, on the whole, is assumed to lead to positive outcomes with respect to job satisfaction, job performance, organizational commitment, and well-being (see also, Kristof, 1996; Daniels and De Jonge, 2010; Kristof-Brown and Guay, 2011). On a more concrete level, two types of P-E fit have been distinguished primarily: the fit between the demands of the environment and the abilities of the person (*demands-abilities fit*) and the fit between the needs of the person and the supplies available in the environment (*needs-supplies fit*; Kristof, 1996; Edwards and Shipp, 2007; Kristof-Brown and Guay, 2011; Krumm et al., 2013). Our line of reasoning is very well-compatible with the latter, as with the concept of needs-supplies fit motivational issues such as “needs, which include biological requisites for survival and psychological desires, motives, and goals” (Edwards and Shipp, 2007, p. 212) and the degree to which “an organization satisfies individuals’ needs, desires, or preferences” (Kristof, 1996, p. 3) are addressed. Needs-supplies fit is supposed to be closely related to “indicators of mental and physical well-being, such as anxiety, depression, tension, and somatic health” (Edwards and Shipp, 2007, p. 225).

A similar notion of P-E fit can be found in the literature on job burnout defined as a “prolonged response to chronic emotional and interpersonal stressors on the job” (Maslach et al., 2001, p. 397). It is postulated that “the greater the perceived incongruity, or mismatch, between the person and the job, the greater the likelihood of burnout” (Maslach and Leiter, 2008, p. 501). Interestingly, however, even though in the research on job burnout a strong case is made for integrating both individual and situational factors, “rather than considering them in separate either-or terms,” and a model of person–environment fit is said “to be an appropriate framework for understanding burnout” (Maslach et al., 2001, p. 413), to the best of our knowledge, there are no studies so far in which an individual’s needs and the environmental supplies were directly measured, and then related to indicators of burnout and physical health. Rather, in studies on job burnout, “the individual’s appraisal of the extent of congruency between him/herself and the job” (Maslach and Leiter, 2008, p. 501) has typically been assessed with the *Areas of Worklife Scale* (AWS; Leiter and Maslach, 2004). Using items as, for example, “I have enough time to do what’s important in my job” (workload), or “Working here forces me to compromise my values” (values), an individual’s general appraisal of six areas of worklife (i.e., workload, control, reward, community, fairness, and values) is determined. The exact nature of the person–environment interaction remains ambiguous, though. First, it is unclear whether congruency between person and the job rests on the person or the environment or both. For example, with respect to the workload item mentioned above, agreeing with it very well could mean that either the job is little demanding or the employee disposes of extraordinary skills that enables him/her to accomplish highly challenging work duties in a comfortable work rhythm. Second, it seems that, in the AWS, both, needs-supplies fit and a demands-abilities fit, are addressed and aggregated, which are hypothesized to have a differential impact on relevant outcome variables, though (Edwards and Shipp, 2007). Third, and more generally, research on needs-supplies fit is plagued by a lack of a theoretical account specifying core needs and respective environmental characteristics suitable to satisfy these needs (Edwards and Shipp, 2007, p. 216).

With our study, we intend to contribute to a clarification of the P-E fit concept in several ways. First, we focus on needs-supplies fit. Second, we will employ direct measures of individuals’ needs and environmental supplies allowing for an analysis of different P-E constellations. Third, and most important, our line of reasoning and the operationalizing of the core constructs is grounded in McClelland’s (1985) theory of implicit motives (for a recent summary, see Schultheiss and Brunstein, 2010), one of the most influential motivational need theories that has lately received increasing interest in the organizational literature as well (van Emmerik et al., 2010; Kazén and Kuhl, 2011; Lang et al., 2012; Uhlmann et al., 2012; Krumm et al., 2013).

### Implicit Affiliation and Power Motives

Implicit motives are defined as “enduring non-conscious needs that drive humans’ behavior toward the attainment of specific classes of incentives” (Schultheiss and Brunstein, 2010, p. ix). More concretely, starting from work by the early pioneers of motive research McClelland et al. (1953), implicit motives are conceived of as dispositional preferences (needs) for specific affective experiences that orient, select, and energize behavior. The *affiliation motive* directs to the establishment and maintenance of positive relationships with others leading to the experience of interpersonal trust, warmth, and belonging. The *power motive* drives an individual to having mental, emotional, or physical impact on other people the affective incentive of which is feeling strong and self-efficacious (Schultheiss, 2008). Implicit motives are not consciously represented and particularly prompt self-initiated, affectively driven behavior when confronted with motive relevant environmental incentives (Spangler, 1992). Consequently, the non-conscious nature of implicit motives implies that they cannot be assessed by self-report. Instead, they are measured with the so-called picture-story exercise (PSE; Schultheiss and Pang, 2007) that builds on the Thematic Apperception Test (TAT) by Morgan and Murray (1935). Implicit motives set the stage for feelings of fulfillment, gratification and happiness through satisfaction of affective needs (Brunstein et al., 1998; Job et al., 2012) and are also closely linked to an individual’s physical well-being (McClelland, 1989; Baumann et al., 2005). On this note, the frustration of implicit motives caused by a lack of opportunities to execute motive driven behavior is detrimental to psychological and physical well-being. The same is true for goal striving that is not backed by a related implicit motive the execution of which then requires continuous and strenuous volitional effort (Kehr, 2004; Baumann et al., 2005; Hofer et al., 2010; Job et al., 2010; Hagemeyer et al., 2013).

### The Present Study

We set out to test the hypothesis that a misfit between the implicit affiliation motive/power motive (needs) and the respective motivational characteristics of one’s job (supplies) is associated with job burnout and physical symptoms. Actually, there are two forms of needs-supplies misfit with respect to implicit motives and the respective environmental incentives. First, an individual might have a strong implicit motive but find herself in a work environment that does not offer the respective incentives resulting in need frustration. Second, an individual might have a weak implicit motive but find himself doing a job that requires respective activities, which are not emotionally satisfying resulting in chronic expenditure of volitional effort to bring oneself to perform these inherently unpleasant occupational activities. Both, need frustration and chronic volitional effort are regarded as “hidden stressors” (Baumann et al., 2005) that impair psychological and physical well-being (Kehr, 2004).

In order to test our hypothesis we sought to survey a sample of full time employed individuals from diverse jobs and companies who concerned themselves with job burnout and, accordingly, approached an internet platform on burnout. They were invited to participate in an online study and asked to take an (unobtrusive) test of implicit affiliation and power motives (Schultheiss and Pang, 2007) and fill in a questionnaire that – in the tradition of work analysis measures (Prümper et al., 1995; Sanchez and Levine, 2001) – characterized in an objective way an array of occupational activities that offer the possibility to satisfy the affiliation (i.e., establishing or maintaining positive relationships with others) or power (i.e., having mental or emotional impact on others) motives, respectively (Schultheiss, 2008). Eventually, participants reported indicators of job burnout and physical well-being. With respect to data analysis, we followed a state-of-the-art procedure using polynomial regression analysis with illustrating results in a response surface analysis (Edwards and Shipp, 2007; Schönbrodt, 2015).

## Materials and Methods

### Participants and Procedure

Participants in the cross-sectional online study were recruited between August and December 2011 by on-line ads at Swiss Burnout^1^, the Swiss national dialog platform on burnout which also provides information and operates a forum for those suffering from burnout. Ads were further published in the news section of the homepage of the Swiss Expert Network on Burnout^2^. The two websites where we had posted our study link were open to a broad public. 427 individuals clicked on our link of whom 175 actually were interested and willing to answer our questionnaire. Of these, 97 participants (39 male, 58 female) were eligible for our analyses as they were employed on a full time basis. We only included full time employees in order to enhance sample homogeneity. They were invited to participate in a study on “situational and individual causes of well-being” and received 20 Swiss Francs (approximately 20 $) for participation. They completed the whole questionnaire. Their average age was 37.77 years (*SD* = 8.92), ranging from 22 to 62. Right after signing in for the study and giving informed consent, participants received access to the online questionnaire in which demographics, implicit motives, job-characteristics, and their well-being were assessed. The study was conducted in compliance with APA ethical standards and the guidelines of the University of Zurich Ethics Commission.

### Measures

#### Implicit Motives

An online-PSE (Bernecker and Job, 2011) was administered to measure participants’ implicit motive dispositions. They were asked to write five imaginative stories based on these picture cues: architect, trapeze artists, women in laboratory, boxer, and nightclub scene (for a sample picture see Supplementary Data Sheet). These pictures are commonly used in research dealing with implicit motives (McClelland, 1975; Smith, 1992; Schultheiss and Brunstein, 2010). The five picture cues were presented in randomized order, in the middle of the screen. Each picture was accompanied by a short instruction saying: “Look at the picture – The program will automatically turn to the next page within 20 s.” On the next page a text field was presented with four reminders about what to include in the story (e.g., “What is happening?” “Who are the people?”). Participants had 5 min to write a story before the program led them to the next picture. They were allowed to proceed to the next picture whenever they were ready regardless of whether full response time had elapsed.

The content of each story was coded according to Winter’s (unpublished manuscript) manual for scoring motive imagery in running text. Affiliation is coded when a sentence describes the establishment, maintenance, or restoration of friendly relationships among persons. Power is scored for content indicating impact or influence on other persons or groups. Each story was coded by two coders who reached an overall agreement of Cohen’s Kappa of 0.92. Scoring disagreements were discussed in full. Participants’ motive scores for affiliation (*M* = 2.78, *SD* = 1.78) and power (*M* = 1.76, *SD* = 1.51) correlated with protocol length (*M* = 337 words, *SD* = 153), *p*s < 0.001. In accordance to Smith et al. (1992) procedure we corrected the raw scores for protocol length by regression and converted them to z scores.

#### Job Characteristics

Due to the lack of an instrument for measuring motive specific job characteristics, we designed a self-report instrument following the best practice of job analysis (e.g., Prümper et al., 1995). Affiliation specific characteristics of the work-place were assessed with five items reflecting how much a job included engagement in friendly social interactions, e.g., “When I’m doing my job, it is necessary to pay attention to others” or “In our team, the social exchange between employees is considered very important” (Cronbach’s α = 0.70). The five items measuring power specific characteristics of the work-place reflected the amount of responsibility for other people as well as the necessity to engage in forceful actions or discussion, e.g., “In my job, I’m responsible for the work done by other employees” or “I frequently have to argue and defend my position in negotiations” (Cronbach’s α = 0.64). Each item was answered on a fully labeled five-point scale ranging from 1 (*not at all true*) to 5 (*extremely true*).

#### Job-Burnout

The German translation of the Maslach Burnout Inventory (Maslach et al., 1996) from Büssing and Glaser (1999) was used to assess the three dimensions: exhaustion (e.g., “I feel emotionally drained from my work.” “I feel used up at the end of the working day.”), cynicism (e.g., “I doubt the significance of my work.” “I just want to do my job and not be bothered.”), and efficacy (e.g., “In my opinion, I am good at my job.” “I have accomplished many worthwhile things in this job.”). The internal consistency of the scale was high (Cronbach’s α = 0.81). Therefore, all items were averaged in an index of job-burnout.

#### Physical Well-Being

A checklist of physical symptoms (Emmons, 1992) including headaches, stomach ache/pain, chest pain, runny/congested nose, coughing/sore throat, faintness/dizziness, out of breath, and stiff/sore muscles was used to asses physical well-being. Participants indicated on a five-point scale (5 *= several times a week, 4 = once per week, 3 = 2–3 times per week, 2 = less frequently, 1 = never*) how frequently they experienced each of the symptoms within the last 2 weeks. The ratings were recoded and averaged in an index of physical symptoms (Cronbach’s α = 0.91).

#### Job Demands

Previous research showed that there is a strong relationship between job demands and burnout, workload, and time pressure explaining about 25–50% of the variance of burnout (Schaufeli and Enzmann, 1998). Research within the framework of the Job-Demands-Resources Model (JD-R) further confirmed that an increase in job demands, such as overload, emotional demands, and work-home interference, predicts burnout by way of a health impairment process (Schaufeli and Bakker, 2004; Schaufeli et al., 2009). Therefore we assessed job-demands using the six items workload subscale from the Areas of Work Life Scale (Leiter and Maslach, 2004). Participants reported their degree of agreement with items such as “I do not have time to do the work that must be done.” and “I work intensely for prolonged periods of time.” using five-point Likert scales (*1 = strongly disagree, 2 = disagree, 3 = hard to decide, 4 = agree, 5 = strongly agree*). The scale was sufficiently reliable (Cronbach’s α = 0.74).

## Results

### Preliminary Analyses

**Table 1** reports the mean, standard deviation, and zero-order correlations among study variables. The affiliation motive was not correlated with any other variable, whereas the power motive was negatively correlated with job demands.

**Table 1:** Descriptive statistics and two-tailed correlations.

	Variable	2	3	4	5	6	7	*M*	*SD*
1	Affiliation motive^1^	0.04	0.00	-0.04	0.07	0.06	0.04	2.78	1.78
2	Power motive^1^		-0.01	-0.08	-0.07	-0.19^+^	-0.22^∗^	1.76	1.51
3	Affiliation job characteristics			0.36^∗∗^	0.04	-0.13	-0.27^∗∗^	3.34	0.69
4	Power job characteristics				0.22^∗^	0.07	0.16	3.33	0.71
5	Burnout					0.74^∗∗^	0.54^∗∗^	2.97	1.21
6	Physical symptoms						0.65^∗∗^	2.27	1.25
7	Job demands							3.37	0.68

*N = 97. ^1^Correlations are computed with motive scores corrected for protocol length, mean, and standard deviation refer to raw motive scores. ^+^p < 0.10; ^∗^p < 0.05; ^∗∗^p < 0.001*.

Affiliation and power specific job characteristics were significantly correlated. This is not surprising, since some sort of social contact at work is the precondition for the possibility to affiliate or have power at work. The two motive specific job characteristics, however, were differentially related to burnout and job demands. Affiliation specific job characteristics were negatively related to job demands whereas power specific job characteristics were positively related to burnout.

As expected, burnout, physical symptoms, and job demands were highly correlated. The more job demands participants faced the higher was their burnout score and the more physical symptoms did they report. These correlations replicate previous research, which shows a consistent relationship of burnout with increased workload (Maslach et al., 2001; Maslach and Leiter, 2008). This is particularly true for the exhaustion dimension of burnout, which then mediates the relationship of workload with cynicism and (reduced) efficacy (Lee and Ashforth, 1996; Demerouti et al., 2001). The relationships found further reflect previous studies on the impact of burnout on physical health where burnout was found to be associated with a variety of somatic symptoms, including sleep disturbances, recurrent headaches, and gastro-intestinal problems (Gorter et al., 2000; Shirom et al., 2005) as well as with an elevated risk of cardiovascular disease (Melamed et al., 1992, 1999).

There were no statistical differences between women and men in implicit motives or job characteristics. However, women reported higher levels of burnout, *t*(95) = -2.76, *p* = 0.007, more physical symptoms, *t*(95) = -2.71, *p* = 0.008, and higher job demands, *t*(95) = -2.00, *p* = 0.048, than men. While previous research is characterized by inconclusive findings on gender effects on burnout, the differences reported here reflect study findings that reveal slightly higher levels of exhaustion among women than among men (Schaufeli and Enzmann, 1998; Ahola et al., 2006) as well as recent reports of health insurance absenteeism data, stating a higher prevalence of the ICD-10 code Z 73.0 (burn-out: state of vital exhaustion) among female health plan members on sick leave (Badura et al., 2011; Kordt and Dak Forschung, 2013). Higher job strain was previously found among female as compared to male burnout patients (Stenlund et al., 2010).

### Testing the Hypothesis: Overview of Analyses

To test the effect of congruence between implicit motives and job specific characteristics we carried out polynomial regressions with response surface analysis, as recommended by Edwards (1994, 2002). This method allows to directly test congruence hypotheses without the well-known disadvantages of difference scores and simple interaction tests (see Kazén and Kuhl, 2011) for a detailed discussion in the context of motive-congruence research. We used the R package for response surface analyses by Schönbrodt (2015). In the polynomial regression an outcome is predicted not only by centered predictors, but also their interaction and the squared values of each.

Hence, the general form of the polynomial regression equation is:

$$Z=b_{0}+b_{1}X+b_{2}Y+b_{3}X^{2}+b_{4}XY+b_{5}Y^{2}+e$$

Z is the dependent variable (i.e., job-burnout, physical symptoms), X is the first predictor (i.e., job characteristics), Y is the second predictor (i.e., motive), b_0_ is the intercept, and b1 to b5 are the estimated coefficients. The results of the polynomial regression are evaluated according to four additional surface test parameters:

a_1_ represents the slope along the “line of congruence,” which is the line of perfect agreement between the two predictors. People who have exactly the same value on each predictor lie on this line. In the graph it is the diagonal line extending from the front to the back corner of the graph. If this parameter is significant it indicates that congruence on one level might be better than congruence on the other level.

a_2_ tells us whether there is a curvature effect of the mean level of the predictors along the line of congruence. If this parameter is significant it indicates that the effect of congruence on an average level differs from the congruence on low or on high level.

a_3_ represents the slope along the “line of incongruence,” from the left to the right corner. It tests for directed difference effects, e.g., the higher the implicit motive as compared to job characteristics the better. In combination with a significant a_4,_ it indicates that there is a lateral shift in the surface away from the line of congruence which might be simply caused by scaling differences between the two predictor variables.

a_4_ is the most important parameter regarding the congruence hypothesis. It tests the curvature along the “line of incongruence” and can answer the question whether an increase in incongruence has any effects on the outcome.

To control for job demands in each analyses standardized residuals from a regression analysis predicting burnout and physical symptoms respectively were used as outcome variables.

### Predicting Job Burnout

First we tested whether incongruence between the affiliation motive and affiliation specific job demands would predict higher levels of job burnout. Therefore, we ran a polynomial regression with job burnout as the outcome and the implicit affiliation motive and affiliation specific job demands as the two predictor variables. The full model was significant, *R*^2^ = 0.135, *p* = 0.020. The bootstrapped coefficients and surface parameters are presented in **Table 2**.

**Table 2:** Parameters from RSA analyses: implicit affiliation motive – affiliation job characteristics discrepancy as predictor of job burnout (*N* = 97).

	*Est*	*SE*	95% CI	β	*p*
b_0_: constant	-0.178	0.135	[-0.44, 0.09]	-0.180	0.189
b_1_: motive	-0.074	0.105	[-0.28, 0.13]	-0.075	0.482
b_2_: job characteristics	0.327	0.108	[0.12, 0.54]	0.331	0.002
b_3_: motive squared	0.162	0.061	[0.04, 0.28]	0.235	0.007
b_4_: motive X job characteristic	-0.141	0.082	[-0.30, 0.02]	-0.172	0.083
b_5_: job characteristic squared	0.017	0.061	[-0.10, 0.14]	0.026	0.777
Surface values					
*a1* = (b_1_ + b_2_)	0.253	0.126	[0.01, 0.50]		0.044
*a2* = (b_3_ + b_4_ + b_5_)	0.038	0.132	[-0.22, 0.30]		0.772
*a3* = (b_1_ - b_2_)	-0.401	0.172	[-0.738, -0.07]		0.019
*a4* = (b_3_ - b_4_ + b_5_)	0.321	0.095	[0.14, 0.51]		0.000

**FIGURE 1:**
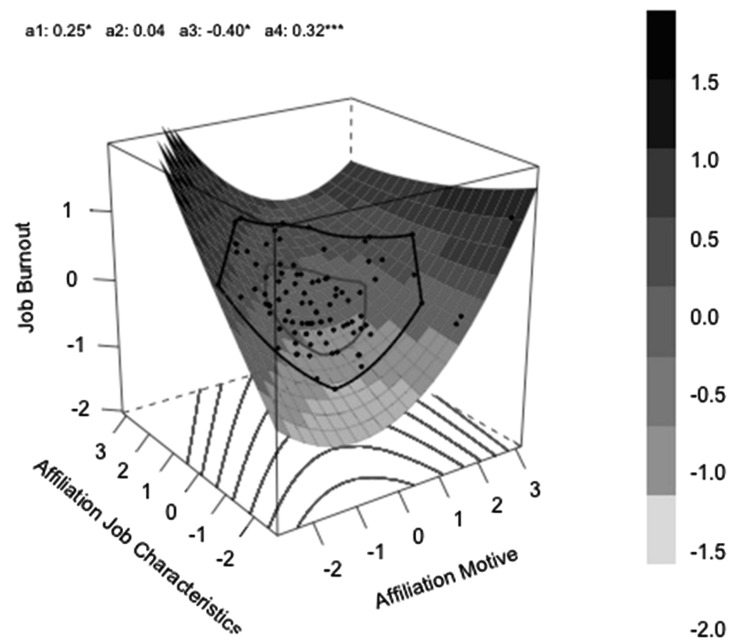
**Job burnout as a function of high or low affiliation motive in interaction with low and high affiliation specific job characteristics**.

Most importantly, the a_4_ surface parameter testing the congruence hypothesis was significant. As predicted, incongruence with respect to the affiliation motive and affiliation specific job characteristics was related to high job burnout (see **Figure 1**). In addition, the surface parameter a_1_ was significant and positive, which indicates that participants who were low in both, the implicit affiliation motive and affiliation specific job demands, reported lower levels of burnout than participants who were congruent on a high level. Finally, the significant a_3_ parameter indicates that there was a lateral shift away from the line of congruence, which indicates a difference in the scaling of the two predictor variables.

Next, we ran the same analyses with the power motive and power specific job demands as predictors of job burnout. The full model did not reach significance, *R*^2^ = 0.095, *p* = 0.101. Although the a_4_ surface parameter was significant, which indicates that at least the pattern corresponds with the congruence hypothesis, we refrain from further interpreting this finding.

### Predicting Physical Symptoms

**FIGURE 2:**
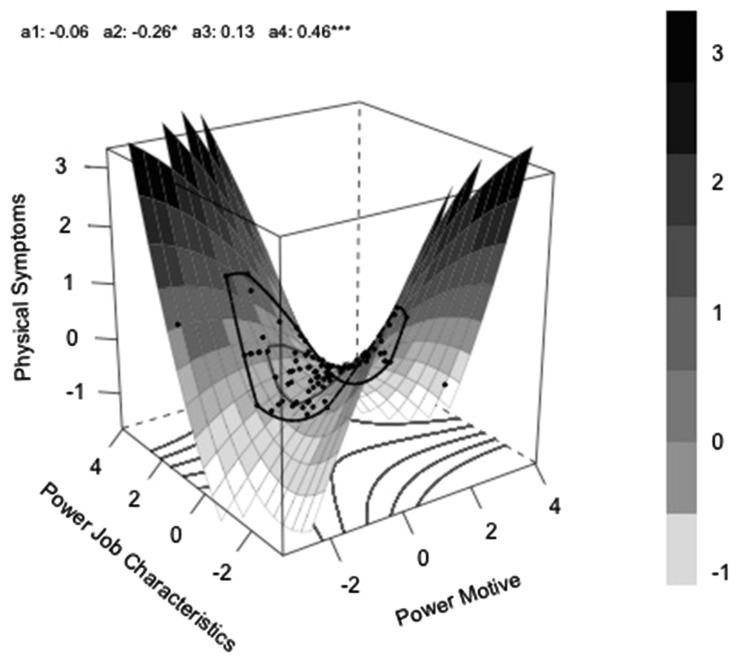
**Physical symptoms as a function of high or low power motive in interaction with low and high power specific job characteristics**.

We ran the same analyses as described above for the affiliation motive and affiliation specific job characteristics and for the power motive and power specific job characteristics predicting physical symptoms as the outcome variable. For affiliation the model did not reach significance, *R*^2^ = 0.085, *p* = 0.147. For power, however, the full model was significant, *R*^2^ = 0.188, *p* = 0.002. The bootstrapped coefficients and surface parameters are presented in **Table 3**. As predicted, the a_4_ surface parameter testing the congruence hypothesis was significant. **Figure 2** shows that incongruence between the power motive and power-specific job characteristics was associated with increased physical symptoms. The only other surface parameter that reached significance was a_2,_ which tests the curvature of the line of congruence. The negative value of the parameter indicates that congruence on a high or low level was related to lowest levels of physical symptoms as compared to congruence on the medium level.

**Table 3:** Parameters from RSA analyses: power motive – power job characteristics discrepancy as predictor of physical symptoms (*N* = 97).

	*Est*	*SE*	95% CI	β	*p*
b_0_: constant	-0.127	0.133	[-0.39, 0.13]	-0.123	0.340
b_1_: motive	0.038	0.076	[-0.11, 0.04]	0.039	0.616
b_2_: job characteristics	-0.093	0.101	[-0.29, 0.10]	-0.094	0.352
b_3_: motive squared	-0.096	0.036	[-0.17, -0.03]	-0.167	0.007
b_4_: motive X job characteristic	-0.361	0.075	[-0.51, -0.21]	-0.356	0.000
b_5_: job characteristic squared	0.195	0.067	[0.06, 0.33]	0.270	0.004
Surface values					
*a1* = (b_1_ + b_2_)	-0.055	0.136	[-0.32, 0.21]		0.683
*a2* = (b_3_ + b_4_ + b_5_)	-0.261	0.124	[-0.51, -0.02]		0.036
*a3* = (b_1_ - b_2_)	0.132	0.116	[-0.10, 0.36]		0.257
*a4* = (b_3_ - b_4_ + b_5_)	0.460	0.093	[0.28, 0.64]		0.000

## Discussion

This research contributes to a finer grained analysis of the needs-supplies fit (Edwards and Shipp, 2007) as core motivational process at the workplace. More concretely, we showed that, as predicted, a motivational incongruence in the affiliative domain (high affiliation motive/low affiliation job characteristics or low affiliation motive/high affiliation job characteristics) was associated with a higher degree of job burnout in comparison to motivational congruence in the domain of affiliation. In the same vein, motivational incongruence in the power domain (high power motive/low power job characteristics or low power motive/high power job characteristics) related to a higher degree of physical symptoms in comparison to motivational congruence in the power domain.

Contrary to our expectations, however, motive incongruence in the affiliation domain was not associated with a higher degree of physical symptoms than affiliation motive congruence, and motive incongruence in the power domain was not associated with a higher degree of job burnout than power motive congruence. Thus, possibly motivational incongruences in the affiliation and power domains are distinctly related to job burnout and physical symptoms. Regarding the power motive, our results coincide with evidence that an inhibited power motive (i.e., a strong power motive that cannot be expressed and thus is frustrated chronically) is associated with heightened physiological arousal, high blood pressure and impaired immune functioning (McClelland, 1979; McClelland and Jemmott, 1980). In addition, it is well-conceivable that the different hormonal basis of affiliation and power motives (Schultheiss et al., 2004; Schultheiss, 2007) is responsible for their differential link to psychological well-being and physical symptoms. Nevertheless, any conclusion about the differential effect of affiliation and power misfit are premature at this time and await replication in future studies with more diverse samples as our sample possibly was somewhat biased toward participants experiencing burnout symptoms (with possible implications for the systematic variance on the study concepts).

Notably, the present data strengthen our assumption of a non-directional person–environment misfit effect, that is, both forms of person–environment misfit comparably compromise well-being and health. More concretely, on the one hand, a strong implicit affiliation or power motive that cannot be satisfied in the work environment due to a lack of respective occupational tasks such as, for example, being in a friendly exchange with other people (affiliation) or taking responsibility for others and influencing on them (power) was related to negative outcomes. On the other hand, people with a weak implicit affiliation or power motive, who were obliged to execute respective occupational activities reported higher degree of job burnout and more physical symptoms. This pattern of results is in contrast to the theoretical assumption of a directional influence forwarded by Langens and McClelland (1997; cf. Kazén and Kuhl, 2011). These authors posit that executing a behavior without gaining pleasure from doing so (i.e., occupational duties that are not backed by a strong respective motive) is more damaging to well-being than a lack of behavior which would give rise to positive affect (i.e., a strong implicit motive without the possibility to execute respective occupational activities). In our view, both forms of the “hidden stressor” (Baumann et al., 2005), that is, need frustration and chronic expenditure of volitional effort, should create inner tension and negative affect, which, in the long run, should be associated with emotional exhaustion – a core characteristic of job burnout.

Our study widens the scope of research on implicit motives in several important ways. First, we add to the still scarce empirical evidence that implicit motives unfold their impact depending on the environmental incentive conditions. For example, Andrews (1967) showed in one of the rare studies on the topic that individual advancement in a firm depended on the fit between the strength of individuals’ achievement or power motives, respectively, and the respective values promoted in a company. More concretely, in a firm emphasizing achievement values, individual advancement (i.e., frequency of promotions and raises) positively related to the strength of the executive’s implicit achievement motive. On the contrary, in a second firm that valued power striving, not only was this relationship reversed (high implicit achievement motive managers were promoted the least), but highly power motivated managers advanced more than managers with a weak implicit power motive (see also Jenkins, 1987 for evidence on the achievement person–environment fit).

Second, existing studies on the interaction of implicit motives and environmental characteristics focused on behavioral outcomes (e.g., performance; Andrews, 1967; Jenkins, 1987; Brunstein and Maier, 2005) while our data clearly show that it also influences affective well-being and health. This parallels evidence from the growing literature on incongruence between implicit and explicit motives and its detrimental effect on psychological and physical well-being (e.g., Brunstein et al., 1998; Hofer and Chasiotis, 2003; Kehr, 2004; Baumann et al., 2005; Langens, 2007; Schüler et al., 2008; Schultheiss et al., 2008; Hofer et al., 2010; Kazén and Kuhl, 2011; Hagemeyer et al., 2013). Evidently, implicit motives are at the very center of emotional life and successful goal striving.

Third, there is a growing interest among I/O-psychologists in implicit motives at the workplace, which allows for putting to test theoretical accounts from basic research in an applied context. As Uhlmann et al. (2012, p. 554) posit “organizational scholars have largely underutilized a highly impactful discovery […]: non-conscious processes and the implicit measures developed to capture them. Despite their limited use, implicit measures hold great promise for organizational research because many phenomena of interest operate outside employees’ complete awareness and control.” Indeed, implicit motives do operate outside of complete awareness and at the same time are an important linchpin of self-regulation. They orient an individual toward not verbally explicated cues inherent in certain behaviors that potentially are apt for satisfying the respective motive (Schultheiss, 2008). Moreoever, satisfaction or frustration of implicit motives has a tremendous impact on affective well-being (for a summary, Schultheiss, 2008; Schultheiss and Brunstein, 2010). Thus, it is no wonder that I/O-psychologist become more and more interested in this fundamental processes of motivated behavior at the workplace (cf. also Kooij et al., 2011; Hertel et al., 2013; Steinmann et al., 2015).

In addition, our study contributes variously to advancing research on stress and burnout. First, it strengthens the evidence on the recent trend toward conceptualizing burnout as resulting from a misfit in the relationship between the employee and his/her work context (Maslach and Leiter, 2008; Brom et al., 2015). While previous research on burnout and occupational health relied solely on self-assessment of work-related person-environment-fit using scales measuring the *perceived* fit regarding six strategic domains of job characteristics (Leiter and Maslach, 2011) or basic need satisfaction and psychological need frustration at work (Trépanier et al., 2015), our study independently measures both the demand and supply side of the equation: to our knowledge, this is the first study demonstrating the association of an actual P-E-fit variable with burnout and physical complaints. We assessed implicit affiliation/power needs and affiliation/power specific job characteristics, and used the congruence between implicit motives and job characteristics as an independent variable in testing its effect on employee health outcomes.

Second, we demonstrated a differential effect of motivational incongruence on employee well-being: while a misfit with respect to an unfulfilled or overstrained affiliation motive at work was associated with a higher degree of burnout, those experiencing a motivational discrepancy in the power domain more frequently reported physical symptoms. These findings may contribute to throwing more light on the mechanisms behind the contribution of new sources of stress typical in today’s globalized, highly competitive, dynamic and efficiency-driven economy such as a lack of reciprocity in the organizational exchange relationship (Sanz, 2008; Piccoli and De Witte, 2015) or illegitimate tasks infringing one’s professional self-image (Semmer et al., 2015) to a growing concern about mental health in the workplace (Wilkerson, 2005, 2007).

### Limitations and Future Directions

Several issues are to be addressed that limit the explanatory power of our data but at the same time open interesting avenues for future research. First, our measures are based on self-report. In future studies, one additionally might want to include a more objective assessment of the job characteristics, for example, by having the supervisor and/or colleagues rate the motivational characteristics of an individual’s occupation. One might also want to assess additional health parameters (e.g., such as sick leaves, number of health center visits). A still finer grained analysis of the health consequences of motivational incongruence would need to assess physiological stress parameters (e.g., saliva cortisol, blood pressure, or heart rate) across a certain period while differing instances of needs-supplies fit would occur.

Second, the cross-sectional design of our study precludes causal inferences. Hence, in future studies one definitely would have to follow a longitudinal design with repeated measures over a sufficiently long period of time so that the two forms of motivational incongruence (i.e., chronic frustration of affective needs and expenditure of volitional effort) would have existed long enough to show the respective psychological and physical consequences.

Third, the sample of the present study was comparatively small limiting statistical power of the analyses. Moreover, our sample consists of individuals who addressed themselves to an online platform that provides information with respect to job burnout. Presumably, these individuals in some way or the other felt affected by some work related concerns. However, it is open to further research whether our results can be generalized to employees more seriously affected by burnout. In this respect, one might want to investigate a (larger) sample of in-patients who are hospitalized for burnout.

Fourth, future studies would have to inspect more closely the continuum between incongruence and congruence. It is well-conceivable that the degree of incongruence causing troubles interacts with individuals’ stress coping capabilities (e.g., Kehr, 2004) and with more general stress inducing conditions such as organizational climate (e.g., Grandey et al., 2012; Giorgi et al., 2016) or general economic crisis (e.g., Mucci et al., 2016).

### Practical Implications

Besides its theoretical contribution, our study has important practical implications. Burnout is a phenomenon widely observed in Western societies. For example, the prevalence rate in the US is 27.8% (Shanafeldt et al., 2012), and in Switzerland it is about 25% (Grebner et al., 2010; Igic et al., 2014). In many cases employees affected by burnout and, more generally, by psychological or physical impairments are absent from the workplace for some period of time causing enormous costs for both the employer and the public health care system. Trying to counteract motivational incongruence seems an easy to implement strategy to remedy this pressing problem. A starting point could be the prevention of motivational incongruence in personnel selection. One could try to achieve a good fit between employees’ implicit motives and the characteristics of an aspired job by assigning employees to jobs according to their implicit motives, be it changing the job demands in order to fit best the motives of the employee (i.e., job design; Taris et al., 2003; Semmer, 2006; Awa et al., 2010; Parker, 2014). The same interventions might be helpful in remedying an already existing motivational incongruence. Another strategy could be the so called “job crafting” by employees (Tims and Bakker, 2010). Job crafting is defined as “a specific form of proactive behavior in which the employee initiates changes in the level of job demands and job resources. […] to fit their jobs to their personal knowledge, skills and abilities on the one hand and to their preferences and needs on the other hand” (Tims and Bakker, 2010, p. 1; Tims et al., 2013). For example, a highly affiliation motivated employee might handle her duties in a more collaborative way and try to find ways to do more teamwork.

Moreover, expanding the unit of analysis from the individual to work units and entire organizations will be one important strategy (Maslach et al., 2012), especially in planning and evaluating interventions toward an optimal placement of individuals in line not only with their capabilities, but also with their basic and psychological needs. Besides, future studies may seek to understand the role of social systems in the workplace in the unfolding of burnout phenomena (Maslach et al., 2012). These would focus on environmental incentives provided by organizational entities and their interplay with individual motive constellations among their members, using multi-level modeling. Should an organizational check-up reveal that the majority of team members are driven by motives which are not satisfied by the current organizational context, person-environment fit could be improved by a combination of corporate culture development and capacity building interventions.

## Conclusion

Our study, on a general level, contributes to a thriving research on implicit motives (Schultheiss and Brunstein, 2010), and, on a more specific level, to a person–environment fit analysis of core work related outcomes, such as burnout and health (Maslach and Leiter, 1997, 2008; Edwards and Shipp, 2007). Even more important, our results hint at potential interventions for preventing or remedying a lack of needs-supplies fit and reduce the risk of impairments of well-being.

## Author Contributions

VB and VJ together formulated the theoretical research question, compiled the materials, and supervised data collection. VB wrote the theoretical introduction, methods and the discussion section, VJ wrote the results section. BS as an burnout expert gave valuable advice in recruiting the sample and contributed to the paper in a substantial way by commenting on an early version of the paper and rewriting certain parts of the discussion.

## Conflict of Interest Statement

The authors declare that the research was conducted in the absence of any commercial or financial relationships that could be construed as a potential conflict of interest.

## Footnotes

1 www.swiss-burnout.ch

2 www.burnoutexperts.ch/news

## References

[B1] Ahola K. Honkonen T. Isometsö E. Kalimo R. Nykyri E. Koskinen S. (2006). Burnout in the general population. *Soc. Psychiatry Psychiatr. Epidemiol.* 41 11–17. 10.1007/s00127-005-0011-5 16341826

[B2] Andrews J. D. W. (1967). The achievement motive and advancement in two types of organizations. *J. Pers. Soc. Psychol.* 6 163–168. 10.1037/h0024689 6035308

[B3] Atkinson J. W. (1957). Motivational determinants of risk-taking behavior. *Psychol. Rev.* 64 359–372. 10.1037/h0043445 13505972

[B4] Awa W. L. Plaumann M. Walter U. (2010). Burnout prevention: a review of intervention programs. *Patient Educ. Couns.* 78 184–190. 10.1016/j.pec.2009.04.008 19467822

[B5] Badura B. Ducki A. Schröder H. Klose J. Macco K. (2011). *Fehlzeiten-Report 2011. Zahlen, Daten, Analyse aus allen Branchen der Wirtschaft. Schwerpunkt: Führung und Gesundheit.* Heidelberg: Springer.

[B6] Bargh J. A. Gollwitzer P. M. Oettingen G. (2010). “Motivation,” in *Handbook of Social Psychology*, eds Fiske S. T. Gilbert D. T. Gardner L. (Hoboken, NJ: John Wiley and Sons Inc), 268–316.

[B7] Baumann N. Kaschel R. Kuhl J. (2005). Striving for unwanted goals: stress-dependent discrepancies between explicit and implicit achievement motives reduce subjective well-being and increase psychosomatic symptoms. *J. Pers. Soc. Psychol.* 89 781–799. 10.1037/0022-3514.89.5.781 16351368

[B8] Beckmann J. Heckhausen H. (2008). “Situational determinants of behavior,” in *Motivation and Action*, eds Heckhausen J. Heckhausen H. (New York, NY: Cambridge University Press), 69–98.

[B9] Bernecker K. Job V. (2011). Assessing implicit motives with an online version of the picture story exercise. *Motiv. Emot.* 35 251–266. 10.1007/s11031-010-9175-8

[B10] Brom S. S. Buruck G. Horvátha I. Richter P. Leiter M. P. (2015). Areas of worklife as predictors of occupational health – A validation study in two German samples. *Burn. Res.* 2 60–70. 10.1016/j.burn.2015.05.001

[B11] Brunstein J. C. Maier G. W. (2005). Implicit and self-attributed motives to achieve: two separate but interacting needs. *J. Pers. Soc. Psychol.* 89 205–222. 10.1037/0022-3514.89.2.205 16162054

[B12] Brunstein J. C. Schultheiss O. C. Grässmann R. (1998). Personal goals and emotional well-being: the moderating role of motive dispositions. *J. Pers. Soc. Psychol.* 75 494–508. 10.1037/0022-3514.75.2.494 9731321

[B13] Büssing A. Glaser J. (1999). *Deutsche Fassung des Maslach Burnout Inventory–General Survey (MBI-GS-D).* Munich: München, Technische Universität, Lehrstuhl für Psychologie.

[B14] Daniels K. De Jonge J. (2010). Match making and match breaking: the nature of match within and around job design. *J. Occup. Organ. Psychol.* 83 1–16. 10.1348/096317909X485144

[B15] Demerouti E. Bakker A. B. Nachreiner F. Schaufeli W. B. (2001). The job-demands-resources model of burnout. *J. Appl. Psychol.* 86 499–512. 10.1037/0021-9010.86.3.499 11419809

[B16] Edwards J. R. (1994). The study of congruence in organizational behavior research: critique and a proposed alternative. *Organ. Behav. Hum. Decis. Process.* 58 51–100. 10.1006/obhd.1994.1029

[B17] Edwards J. R. (2002). “Alternatives to difference scores: polynomial regression analysis and response surface methodology,” in *Advances in Measurement and Data Analysis*, eds Drasgow N. F. Schmitt N. W. (San Francisco, CA: Jossey-Bass), 350–400.

[B18] Edwards J. R. Shipp A. J. (2007). “The relationship between person-environment fit and outcomes: an integrative theoretical framework,” in *Perspectives on Organizational Fit*, eds Ostroff C. Judge T. A. (New York, NY: Lawrence Erlbaum), 209–258.

[B19] Emmons R. A. (1992). Abstract versus concrete goals: personal striving level, physical illness, and psychological well-being. *J. Pers. Soc. Psychol.* 62 292–300. 10.1037/0022-3514.62.2.292 1556661

[B20] Giorgi G. Mancuso S. Fiz Perez F. Castiello D. Antonio A. Mucci N. (2016). Bullying among nurses and its relationship with burnout and organizational climate. *Int. J. Nurs. Pract.* 22 160–168. 10.1111/ijn.12376 25825025

[B21] Gorter R. C. Eijkman M. A. J. Hoogstraten J. (2000). Burnout and health among Dutch dentists. *Eur. J. Oral Sci.* 108 261–267. 10.1034/j.1600-0722.2000.108004261.x 10946759

[B22] Grandey A. Foo S. C. Groth M. Goodwin R. E. (2012). Free to be you and me: a climate of authenticity alleviates burnout from emotional labor. *J. Occup. Health Psychol.* 17 1–14. 10.1037/a0025102 21875210

[B23] Grebner S. Berlowitz I. Alvarado V. Cassina M. (2010). *Stressstudie 2010. Stress bei Schweizer Erwerbstätigen. Zusammenhänge zwischen Arbeitsbedingungen, Personenmerkmalen, Befinden und Gesundheit.* Bern: Staatssekretariat für Wirtschaft (SECO).

[B24] Hagemeyer B. Neberich W. Asendorpf J. B. Neyer F. J. (2013). (In) Congruence of implicit and explicit communal motives predicts the quality and stability of couple relationships. *J. Personal.* 81 390–402. 10.1111/jopy.12016 23072495

[B25] Hertel G. Thielgen M. Rauschenbach C. Grube A. Stamov-Rossnagel C. Krumm S. (2013). “Age differences in motivation and stress at work,” in *Age-Differentiated Work Systems*, eds Schlick C. Frieling E. Wegge J. (Berlin: Springer-Verlag), 119–147.

[B26] Hofer J. Busch H. Bond M. H. Li M. Law R. (2010). Is motive-goal congruence in the power domain beneficial for individual well-being? An investigation in a German and two Chinese samples. *J. Res. Pers.* 44 610–620. 10.1016/j.jrp.2010.08.001

[B27] Hofer J. Chasiotis A. (2003). Congruence of life goals and implicit motives as predictors of life satisfaction: cross-cultural implications of a study of Zambian male adolescent. *Motiv. Emot.* 27 251–272. 10.1023/A:1025011815778

[B28] Hull C. L. (1943). *Principles of Behavior: An Introduction to Behavior Theory.* New York, NY: Appleton-Century Crofts, Inc.

[B29] Igic I. Keller A. Brunner B. Wieser S. Elfering A. Semmer N. (2014). *Job-Stress-Index 2014. Erhebung von Kennzahlen zu psychischer Gesundheit und Stress bei Erwerbstätigen in der Schweiz.* Gesundheitsförderung Schweiz Arbeitspapier 26 Bern: Gesundheitsförderung Schweiz.

[B30] Jenkins S. R. (1987). Need for achievement and women’s careers over 14 years: evidence for occupational structure effects. *J. Pers. Soc. Psychol.* 53 922–932. 10.1037/0022-3514.53.5.922 3681657

[B31] Job V. Bernecker K. Dweck C. S. (2012). Are implicit motives the need to feel certain affect? Motive-affect congruence predicts relationship satisfaction. *Pers. Soc. Psychol. Bull.* 38 1552–1565. 10.1177/0146167212454920 22854792

[B32] Job V. Oertig D. Brandstätter V. Allemand M. (2010). Discrepancies between implicit and explicit motivation and unhealthy eating behavior. *J. Pers.* 78 1209–1238. 10.1111/j.1467-6494.2010.00648.x 20545817

[B33] Kazén M. Kuhl J. (2011). Directional discrepancy between implicit and explicit power motives is related to well-being among managers. *Motiv. Emot.* 35 317–327. 10.1007/s11031-011-9219-8

[B34] Kehr H. M. (2004). Implicit/explicit motive discrepancies and volitional depletion among managers. *Pers. Soc. Psychol. Bull.* 30 315–327. 10.1177/0146167203256967 15030623

[B35] Kooij D. T. De Lange A. H. Jansen P. G. Kanfer R. Dikkers J. S. (2011). Age and work-related motives: results of a meta-analysis. *J. Organ. Behav.* 32 197–225. 10.1002/job.665

[B36] Kordt M. Dak Forschung (2013). *Gesundheitsreport 2013. Analyse der Arbeitsunfähigkeitsdaten. Update Psychische Erkrankungen - Sind wir Heute Anders Krank?.* Hamburg: DAK Gesundheit and IGES.

[B37] Kristof A. L. (1996). Person-organization fit: an integrative review of its conceptualizations, measurement, and implications. *Pers. Psychol.* 49 1–49. 10.1111/j.1744-6570.1996.tb01790.x

[B38] Kristof-Brown A. L. Guay R. P. (2011). “Person– environment fit,” in *American Psychological Association Handbook of Industrial and Organizational Psychology*, Vol. 3 ed. Zedeck S. (Washington, DC: American Psychological Association), 1–50.

[B39] Krumm S. Grube A. Hertel G. (2013). No time for compromises: age as a moderator of the relation between needs–supply fit and job satisfaction. *Eur. J. Work Organ. Psychol.* 22 547–562. 10.1080/1359432X.2012.676248

[B40] Lang J. W. Zettler I. Ewen C. Hülsheger U. R. (2012). Implicit motives, explicit traits, and task and contextual performance at work. *J. Appl. Psychol.* 97 1201–1217. 10.1037/a0029556 22867444

[B41] Langens T. McClelland D. C. (1997). Implicit motives, explicit motives, and emotional well-being. *Poster Presented at the 105th Convention of the American Psychological Association*, Chicago, IL.

[B42] Langens T. A. (2007). Congruence between implicit and explicit motives and emotional well-being: the moderating role of activity inhibition. *Motiv. Emot.* 31 49–59. 10.1007/s11031-006-9038-5

[B43] Lee R. T. Ashforth B. E. (1996). A meta-analytic examination of the correlates of the three dimensions of burnout. *J. Appl. Psychol.* 81 123–133. 10.1037/0021-9010.81.2.123 8603909

[B44] Leiter M. P. Maslach C. (2004). “Areas of Worklife: a structured approach to organizational predictors of job burnout,” in *Research in Occupational Stress and Well-Being*, Vol. 3 eds Perrewe P. L. Ganster D. C. (Oxford: Elsevier), 91–134.

[B45] Leiter M. P. Maslach C. (2011). *Areas of Worklife Survey. Manual and Sampler Set*, 5th Edn. Menlo Park, CA: Mind Garden.

[B46] Lewin K. (1951). “Behavior and development as a function of the total situation,” in *Field Theory in Social Science: Selected Theoretical Papers by Kurt Lewin*, ed. Cartwright D. (New York, NY: Harper), 238–303.

[B47] Maslach C. Jackson S. E. Leiter M. P. (1996). *Maslach Burnout Inventory Manual*, 3rd Edn. Palo Alto, CA: Consulting Psychologist Press.

[B48] Maslach C. Leiter M. P. (1997). *The Truth about Burnout.* San Francisco, CA: Jossey Bass.

[B49] Maslach C. Leiter M. P. (2008). Early predictors of job burnout and engagement. *J. Appl. Psychol.* 93 498–512. 10.1037/0021-9010.93.3.498 18457483

[B50] Maslach C. Leiter M. P. Jackson S. E. (2012). Making a significant difference with burnout interventions: researcher and practitioner collaboration. *J. Organ. Behav.* 33 296–300. 10.1002/job.784

[B51] Maslach C. Schaufeli W. B. Leiter M. P. (2001). “Job burnout,” in *Annual Review of Psychology*, Vol. 52 eds Fiske S. T. Schacter D. L. Zahn-Waxler C. (Palo Alto, CA: Annual Reviews), 397–422. 10.1146/annurev.psych.52.1.397 11148311

[B52] McClelland D. C. (1975). *Power: The Inner Experience.* New York, NY: Irvington.

[B53] McClelland D. C. (1979). Inhibited power motivation and high blood pressure in men. *J. Abnorm. Psychol.* 88 182–190. 10.1037/0021-843X.88.2.182 447901

[B54] McClelland D. C. (1985). How motives, skills, and values determine what people do. *Am. Psychol.* 40 812–825. 10.1037/0003-066X.40.7.812

[B55] McClelland D. C. (1989). Motivational factors in health and disease. *Am. Psychol.* 44 675–683. 10.1037/0003-066X.44.4.675 2729741

[B56] McClelland D. C. Atkinson J. W. Clark R. A. Lowell E. L. (1953). *The Achievement Motive.* New York, NY: Appleton-Century-Crofts.

[B57] McClelland D. C. Jemmott J. B. (1980). Power motivation, stress, and physical illness. *J. Hum. Stress* 6 6–15. 10.1080/0097840X.1980.9934531 7451955

[B58] Melamed S. Shirom A. Froom P. (1992). Burnout and risk factors for cardiovascular diseases. *Behav. Med.* 18 53–61. 10.1080/08964289.1992.9935172 1392214

[B59] Melamed S. Ugarten U. Shirom A. Kahana L. Lerman Y. Froom P. (1999). Chronic burnout, somatic arousal and elevated salivary cortisol levels. *J. Psychosom. Res.* 46 591–598. 10.1016/S0022-3999(99)00007-0 10454175

[B60] Morgan C. Murray H. A. (1935). A method for investigating fantasies: the thematic apperception test. *Arch. Neurol. Psychiatry* 34 289–306. 10.1001/archneurpsyc.1935.02250200049005

[B61] Mucci N. Giorgi G. Roncaioli M. Fiz Perez J. Arcangeli G. (2016). The correlation between stress and economic crisis: a systematic review. *Neuropsychiatr. Dis. Treat.* 12 983–993. 10.2147/NDT.S98525 27143898 PMC4844458

[B62] Parker S. K. (2014). Beyond motivation: job and work design for development, health, ambidexterity, and more. *Annu. Rev. Psychol.* 65 661–691. 10.1146/annurev-psych-010213-115208 24016276

[B63] Piccoli B. De Witte H. (2015). Job insecurity and emotional exhaustion: testing psychological contract breach versus distributive injustice as indicators of lack of reciprocity. *Work Stress* 29 246–263. 10.1080/02678373.2015.1075624

[B64] Prümper J. Hartmannsgruber K. Frese M. (1995). KFZA. Kurzfragebogen zur Arbeitsanalyse [A short questionnaire for job analysis]. *Z. Arbeits Organisationspsychol.* 39 125–132.

[B65] Sanchez J. Levine E. (2001). “The analysis of work in the 20th and 21st centuries,” in *Handbook of Industrial, Work and Organizational Psychology: Personnel Psychology*, Vol. 1 eds Anderson N. Ones D. S. Sinangil H. K. Viswesvaran C. (London: SAGE Publications Ltd.), 71–90.

[B66] Sanz A. (2008). Burnout als Gruppenphänomen. Ein soziologisch –gruppendynamischer Beitrag zum Wandel der (Team-)arbeit. *Gruppendynam. Organ.* 1 88–106.

[B67] Schattke K. Brandstätter V. Taylor G. Kehr H. M. (2014). Flow on the rocks: motive-incentive congruence enhances flow in rock climbing. *Int. J. Sport Psychol.* 45 603–620. 10.7352/IJSP2014.45.603

[B68] Schaufeli W. B. Bakker A. B. (2004). Job demands, job resources, and their relationship with burnout and engagement: a multi-sample study. *J. Organ. Behav.* 25 203–315. 10.1002/job.248

[B69] Schaufeli W. B. Bakker A. B. van Rhenen W. (2009). How changes in job demands and resources predict burnout, work engagement, and sickness absenteeism. *J. Organ. Behav.* 30 893–917. 10.1002/job.595

[B70] Schaufeli W. B. Enzmann D. (1998). *The Burnout Companion to Research and Practice. A Critical Analysis.* London: Taylor and Francis.

[B71] Scheffer D. Heckhausen H. (2008). “Trait theories of motivation,” in *Motivation and Action*, eds Heckhausen J. Heckhausen H. (New York, NY: Cambridge University Press), 42–68.

[B72] Schönbrodt F. D. (2015). *RSA: An R Package for Response Surface Analysis (Version 0.9.8).* Available at: http://cran.r-project.org/web/packages/RSA/index.html

[B73] Schüler J. Job V. Fröhlich S. M. Brandstätter V. (2008). A high implicit affiliation motive does not always make you happy: a corresponding explicit motive and corresponding behavior are further needed. *Motiv. Emot.* 32 231–242. 10.1007/s11031-008-9096-y

[B74] Schultheiss O. C. (2007). “A biobehavioral model of implicit power motivation arousal, reward, and frustration,” in *Social Neuroscience: Integrating Biological and Psychological Explanations of Social Behavior*, eds Harmon-Jones E. Winkielman P. (New York, NY: Guilford), 176–196.

[B75] Schultheiss O. C. (2008). “Implicit motives,” in *Handbook of Personality: Theory and Research*, 3rd Edn, eds John O. P. Robins R. W. Pervin L. A. (New York, NY: Guilford), 603–633.

[B76] Schultheiss O. C. Brunstein J. C. (eds) (2010). *Implicit Motives.* New York, NY: Cambridge University Press.

[B77] Schultheiss O. C. Jones N. M. Davis A. Q. Kley C. (2008). The role of implicit motivation in hot and cold goal pursuit: effects on goal progress, goal rumination, and emotional well-being. *J. Res. Personal.* 42 971–987. 10.1016/j.jrp.2007.12.009

[B78] Schultheiss O. C. Pang J. S. (2007). “Measuring implicit motives,” in *Handbook of Research Methods in Personality Psychology*, eds Robins R. W. Fraley R. C. Krueger R. (New York, NY: Guilford), 322–344.

[B79] Schultheiss O. C. Wirth M. M. Stanton S. J. (2004). Effects of affiliation and power motivation arousal on salivary progesterone and testosterone. *Horm. Behav.* 46 592–599. 10.1016/j.yhbeh.2004.07.005 15555501

[B80] Semmer N. K. (2006). Job stress interventions and the organization of work. *Scand. J. Work Environ. Health* 32 515–527. 10.5271/sjweh.1056 17173207

[B81] Semmer N. K. Jacobshagen N. Meier L. L. Elfering A. Beehr T. A. Kälin W. (2015). Illegitimate tasks as a source of work stress. *Work Stress* 29 32–56. 10.1080/02678373.2014.1003996 25892839 PMC4396521

[B82] Shanafeldt T. D. Boone S. Tan L. Dyrbye L. N. Sotile W. Satele D. (2012). Burnout and satisfaction with work-life-balance among US physicians relative to the General US Population. *Arch. Intern. Med.* 172 1377–1385. 10.1001/archinternmed.2012.3199 22911330

[B83] Shirom A. Melamed S. Toker S. Berliner S. Shapira E. (2005). Burnout, mental and physical health: a review of the evidence and a proposed explanatory model. *Int. Rev. Ind. Organ. Psychol.* 20 269–309.

[B84] Smith C. E. (ed.) (1992). *Motivation and Personality. Handbook of Thematic Content Analysis.* Cambridge, MA: Cambridge University Press.

[B85] Smith C. P. Feld S. C. Franz C. E. (1992). “Methodological considerations: steps in research employing content analysis systems,” in *Handbook of Thematic Content Analysis*, ed. Smith C. P. (New York, NY: Cambridge University Press), 515–536.

[B86] Spangler W. D. (1992). Validity of questionnaire and TAT measures of need for achievement: two meta-analyses. *Psychol. Bull.* 112 140–154. 10.1037/0033-2909.112.1.140

[B87] Stanton S. J. Hall J. L. Schultheiss O. C. (2010). “Properties of motive-specific incentives,” in *Implicit Motives*, eds Schultheiss O. C. Brunstein J. C. (New York, NY: Oxford University Press), 245–278.

[B88] Steinmann B. Dörr S. L. Schultheiss O. C. Maier G. W. (2015). Implicit motives and leadership performance revisited: what constitutes the leadership motive pattern? *Motiv. Emot.* 39 167–174. 10.1007/s11031-014-9458-6

[B89] Stenlund T. Ahlgren C. Lindahl B. Burell G. Knutsson A. Stegmayr B. (2010). Patients with burnout in relation to gender and a general population. *Scand. J. Public Health* 35 516–523. 10.1080/14034940701271874 17852977

[B90] Taris T. W. Kompier M. A. J. Geurts S. A. E. Schreurs P. J. G. Schaufeli W. B. de Boer E. (2003). Stress management interventions in the dutch domiciliary care sector: findings from 81 organizations. *Int. J. Stress Manage.* 10 297–325. 10.1037/1072-5245.10.4.297

[B91] Tims M. Bakker A. B. (2010). Job crafting: towards a new model of individual job redesign. *SA J. Ind. Psychol.* 36 1–9. 10.4102/sajip.v36i2.841

[B92] Tims M. Bakker A. B. Derks D. (2013). The impact of job crafting on job demands, job resources, and well-being. *J. Occup. Health Psychol.* 18 230–240. 10.1037/a0032141 23506549

[B93] Trépanier S. G. Forest J. Fernet C. Austin S. (2015). On the psychological and motivational processes linking job characteristics to employee functioning: insights from self-determination theory. *Work Stress* 29 286–305. 10.1080/02678373.2015.1074957

[B94] Uhlmann E. L. Leavitt K. Menges J. I. Koopman J. Howe M. Johnson R. E. (2012). Getting explicit about the implicit: a taxonomy of implicit measures and guide for their use in organizational research. *Organ. Res. Methods* 15 553–601. 10.1177/1094428112442750

[B95] van Emmerik H. Gardner W. L. Wendt H. Fischer D. (2010). Associations of culture and personality with McClelland’s motives: a cross-cultural study of managers in 24 countries. *Group Organ. Manage.* 35 329–367. 10.1177/1059601110370782

[B96] Wilkerson B. (2005). *Roadmap to Mental Health and Excellence at Work in Canada.* Toronto: Global Business and Economic Roundtable on Addiction and Mental Health in the Workplace.

[B97] Wilkerson B. (2007). *The Stress Invasion. The Other Crisis in Climate Change.* Toronto: Global Business and Economic Roundtable on Addiction and Mental Health in the Workplace.

