# Supplementary material for: Motivational Incongruence and Well-Being at the Workplace: Person-Job Fit, Job Burnout, and Physical Symptoms
Source: Front Psychol. 2016 Aug 11;7:1153. doi: 10.3389/fpsyg.2016.01153 (PMC4981689; doi:10.3389/fpsyg.2016.01153)

**Supplementary Material**

Sample Picture („Architect at Desk“) Used in the Picture Story Exercise to Measure Implicit Affiliation and Power Motives (Smith, 1992, p. 634)


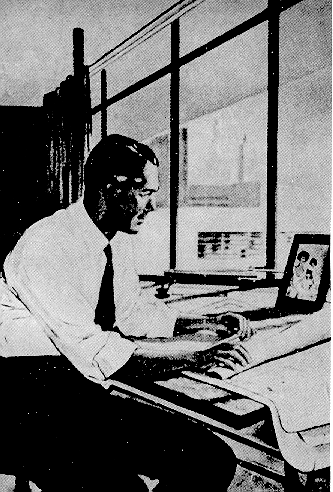

Supplement: Supplementary file 1 [file Data_Sheet_1.DOCX]
